# The CXCR4/CXCR7/CXCL12 Axis Is Involved in a Secondary but Complex Control of Neuroblastoma Metastatic Cell Homing

**DOI:** 10.1371/journal.pone.0125616

**Published:** 2015-05-08

**Authors:** Annick Mühlethaler-Mottet, Julie Liberman, Kelly Ascenção, Marjorie Flahaut, Katia Balmas Bourloud, Pu Yan, Nicolas Jauquier, Nicole Gross, Jean-Marc Joseph

**Affiliations:** 1 Department of Pediatrics, University Hospital CHUV, University of Lausanne, Lausanne, Switzerland; 2 Department of Pathology, University Hospital CHUV, University of Lausanne, Lausanne, Switzerland; Universite de Rennes 1, FRANCE

## Abstract

Neuroblastoma (NB) is one of the most deadly solid tumors of the young child, for which new efficient and targeted therapies are strongly needed. The CXCR4/CXCR7/CXCL12 chemokine axis has been involved in the progression and organ-specific dissemination of various cancers. In NB, CXCR4 expression was shown to be associated to highly aggressive undifferentiated tumors, while CXCR7 expression was detected in more differentiated and mature neuroblastic tumors. As investigated *in vivo*, using an orthotopic model of tumor cell implantation of chemokine receptor-overexpressing NB cells (IGR-NB8), the CXCR4/CXCR7/CXCL12 axis was shown to regulate NB primary and secondary growth, although without any apparent influence on organ selective metastasis. In the present study, we addressed the selective role of CXCR4 and CXCR7 receptors in the homing phase of metastatic dissemination using an intravenous model of tumor cell implantation. Tail vein injection into NOD-scid-gamma mice of transduced IGR-NB8 cells overexpressing CXCR4, CXCR7, or both receptors revealed that all transduced cell variants preferentially invaded the adrenal gland and typical NB metastatic target organs, such as the liver and the bone marrow. However, CXCR4 expression favored NB cell dissemination to the liver and the lungs, while CXCR7 was able to strongly promote NB cell homing to the adrenal gland and the liver. Finally, coexpression of CXCR4 and CXCR7 receptors significantly and selectively increased NB dissemination toward the bone marrow. In conclusion, CXCR4 and CXCR7 receptors may be involved in a complex and organ-dependent control of NB growth and selective homing, making these receptors and their inhibitors potential new therapeutic targets.

## Introduction

Neuroblastoma (NB) is a typical pediatric neoplasm derived from embryonic neural crest cells. The tumor recapitulates characteristics of its originating cells, with an extensive heterogeneity. As a consequence, the disease displays a remarkable clinical diversity, ranging from spontaneous regression or maturation in response to limited treatment, to fatal progression and dissemination to privileged sites, such as bone marrow (BM), bone, lymph nodes and liver, and to the lungs although infrequently [1,2]. As with most cancers, NB progression is extremely difficult to control once disseminated. Residual disease, essentially in the bone and BM, will eventually relapse and progress, despite high doses and multi-modal therapies [3]. Therefore, better understanding of the metastatic process and identification of new therapeutic targets to fight the metastatic disease are strongly needed.

Chemokines and their receptors have been originally described as essential mediators of leukocyte directional migration, and have further emerged as crucial players in all stages of tumor development [4–8]. CXCR4 was described as the most frequently expressed chemokine receptor on tumor cells [9]. As a result of binding to its unique chemokine ligand, CXCL12, CXCR4 is known to activate G protein-mediated signaling cascades, leading to tumor proliferation, survival, angiogenesis, and chemotaxis in multiple cancers [8,10,11]. CXCL12 is expressed at significantly higher level in murine adrenal glands, compared to BM, liver and lungs, while CXCL12 expression is barely detectable in kidney and heart [12,13]. A role for CXCR4 in mediating secondary growth and preferential homing, in response to CXCL12 released by metastatic target organs, remains unclear and controversial [10,14–20]. In particular, we have previously shown in an orthotopic NB xenograft model [21], that CXCR4 mostly promoted NB primary and secondary tumor growth, without influencing organ-specific dissemination of malignant NB cells [13].

CXCR7, an alternate receptor for CXCL12, has been subsequently identified next to CXCR4 and was shown to elicit significant distinct functions and a stronger affinity for CXCL12 [22,23]. Ligand binding to CXCR7 does not mediate CXCR4-like, classical G protein-coupled receptor-mediated calcium mobilization [22–25], but alternative signaling pathways regulating tumor growth and dissemination [26,27]. Thus, contribution of CXCR7 within tumor environment has introduced a new level of complexity to CXCL12 signaling. A role for CXCR7, alone or in association with CXCR4 has been proposed in various solid tumors, including breast, glioma, lung, and prostate cancers [26,27]. Elevated levels of CXCR7 have been detected, particularly in the tumor endothelial cell-associated vasculature [10,28,29]. CXCR7 was also shown to potentiate and regulate trans-endothelial migration of circulating CXCR4^+^CXCR7^+^-tumor cells, thus leading to enhanced extravasation [30,31]. Thus, CXCR7 is suspected to be involved in the regulation of organ-specific metastasis [10,28,32]. However, co-expression of the two CXCL12 receptors may result in the inhibition of invasive properties in response to CXCL12 both *in vitro* and *in vivo* [33], suggesting a possible competing cross-talk between CXCR4 and CXCR7 during metastatic dissemination and homing [30,31].

There is currently very little knowledge on the CXCR4/CXCR7/CXCL12 axis involvement in human NB progression. A previous report described CXCR7 expression in some NB cell lines, and a possible *in vitro* CXCR7-mediated NB migration in presence of CXCL12 produced by mesenchymal cells [34]. Other reports revealed that CXCR4 and CXCR7 expression patterns on NB tumors are distinct or even opposed, as scarce and preferential expression of CXCR7 was observed in neural-associated compartment of differentiated and matured tumors [35], while CXCR4 expression was associated to highly aggressive, undifferentiated tumors [13,36]. Interestingly, aggressive tumors and metastatic NB cell lines were shown to express both CXCR4/CXCR7 [34]. *In vivo* studies performed by implantation of transduced NB cell lines expressing CXCR7, CXCR4, or a combination of both receptors, either subcutaneously or directly in adrenal gland (AG), showed that in contrast to CXCR4, CXCR7 elicited anti-tumorigenic properties, particularly in presence of CXCR4 [35].

These results witnessed a putative CXCL12 receptors cross-talk in NB cell lines, and suggested the implication of the global CXCR7/CXCR4/CXCL12 axis in the regulation of NB progression. However, these studies did not reveal an influence of any CXCL12 receptor on organ-specific metastatic dissemination, particularly in NB preferred sites (such as liver, and bone marrow) [13,35]. Thus the control of NB progression and organ-specific dissemination by the CXCR4/CXCR7/CXCL12 axis remains to be clarified.

In this report we focused our investigations in the particular homing phase of NB cells metastatic dissemination, where the involvement of CXCR4 and CXCR7 receptors was specifically investigated. Thus, we developed an *in vivo* intravenous injection model (iv model), allowing evaluation of site-specific tumor cells seeding, while avoiding initial steps of the metastatic process, such as primary tumor cell detachment and intravasation. By tail vein injection of CXCR4- or/and CXCR7-overexpressing IGR-NB8 cells in immunodeficient NOD-scid-gamma (NSG) mice, we essentially demonstrated that CXCR4 and CXCR7 receptors do influence NB cell homing, but in a complex and organ-specific way. Indeed, CXCR4 preferentially favors NB cell implantation in the liver and the lungs, while CXCR7 enhances seeding to the liver and the AG, and both receptors increase BM invasion.

## Materials and Methods

### Ethics statement

All *in vivo* procedures were performed under the guidelines of the Swiss Animal Protection Ordinance and the Animal Experimentation Ordinance of the Swiss Federal Veterinary Office (FVO). Animal experimentation protocols were approved by the Swiss FVO (authorization number: 1564.6). All reasonable efforts were made to ameliorate suffering, including anesthesia for painful procedures.

### Cell lines

The previously described transduced variants of the human IGR-NB8 cell line [35,37], stably overexpressing individual CXCR4 (NB8x4), CXCR7 (NB8x7), a combination of both receptors (NB8x4x7), or control cells (NB8pMigr) were used in this study. Transduced GFP-expressing cells were sorted by FACS Aria cell sorter (BD Biosciences, San Jose, CA, USA) and cultivated as whole cell populations [35,37]. The NB8x4, NB8x7, NB8x4x7 and NB8pMigr transduced cell lines were cultured in Dubelcco’s modified Eagle’s medium (DMEM) (Gibco, Paisley, UK) supplemented with 1% penicillin/streptomycin (Gibco) and 10% heat inactivated Foetal Bovine Serum (FBS) (Sigma-Aldrich, St Louis, MO, USA). HUVECs were obtained from Lonza (Walkersville, MD, USA), and were cultured on 0.5% gelatin-coated flasks in EGM-2 Medium (EGM-2 Bullet Kit, Lonza, Cologne, Germany) up to passage 7.

### Flow cytometry

Single cells were stained with mouse anti-CXCR4 (clone 12G5, BD Biosciences), or mouse anti-CXCR7 (clone 11G8, R&D systems) antibodies, as previously described [13]. Alexa Fluor 647-labeled goat anti-mouse was used as secondary antibody (Invitrogen, Carlsbad, CA, USA). Ten thousand events were analyzed by Gallios cytometer (Beckman Coulter).

### Adhesion assay

HUVECs were allowed to attach in a 24-well plate (Costar), preliminary coated with 0.5% gelatin (Sigma-Aldrich, St Louis, MO, USA), in EGM-2 Medium (Lonza) at 37°C until confluence. EGFP-expressing NB cells, preliminary starved in serum free medium (SFM) for 12h, were harvested with PBS-5mM EDTA, and wash with PBS. Then, 10^5^ NB cells were seeded on HUVEC monolayer in SFM, in presence or in absence of 100ng/ml CXCL12 (PeproTech, Rocky Hill, NJ, USA) for 1h at 37°C. After two brief wash with PBS, eGFP fluorescence in each well was read using a plate reader (λ_Excitation_ 485 nm; λ_Emission:_ 515 nm). EGFP fluorescence of HUVECs in absence of NB cells was measured as control. Number of adherent NB cells was then assessed according to a standard curve method. Five independent experiments were conducted in triplicates.

### Invasion assay

Growth factor-reduced Matrigel (BD Biosciences), diluted at 3 mg/ml in serum free-DMEM (SFM) and supplemented with 1% N2 supplement (Gibco) (N2-SFM), was gently spread across the entire surface of 8μm porosity-polycarbonate filters of Costar cell culture inserts (24-well format, BD Biosciences). Matrigel was then allowed to solidify for 2h at 37°C. NB cells (5×10^4^), starved in SFM for 12h, were suspended in 100 μl N2-SFM, and seeded in the upper compartment of the insert. The lower compartment was filled with N2-SFM, in presence or in absence of 100 ng/ml CXCL12 (PeproTech). After 48h incubation at 37°C, Matrigel and medium from both upper and lower chambers were carefully removed. Membranes were washed with PBS, and fixed for 10 min in 4% paraformaldehyde (PFA) (Fluka, Buchs, Switzerland). Non-invasive cells were carefully scraped from the upper side of the filter. Membranes were then stained with DAPI, and invasive cells on the lower side were counted in one 10x magnification field per condition using fluorescent microscopy (Leica Microsystems Schweiz AG, Switzerland). Imaging was performed using a camera DFC345 FX (Leica Microsystems Schweiz AG, Switzerland). Three independent experiments were conducted in triplicates.

### Intravenous injection in mice

All animal experiments were carried out with NOD-scid-gamma (NSG) mice from the Jackson Laboratory (UK). All in vivo procedures were performed under the guidelines of the Swiss AnimalProtection Ordinance and the Animal Experimentation Ordinance of the Swiss Federal Veterinary Office (FVO). Animal experimentation protocols were approved by the Swiss FVO (authorization number: 1564.6). All reasonable efforts were made to ameliorate suffering, including anesthesia for tumor cell injections. Transduced IGR-NB8 cells were detached in 5mM PBS-EDTA, 2×10^6^ cells were suspended in 200 μl serum-free DMEM, and injected in the tail vein of 5 to 6 seven-week old mice using a 29G insulin syringes (BD Biosciences). After tumor cell implantations, mice were checked twice a week for their physical conditions, activity, and posture, as well as by palpation for tumor detection. Humane endpoints were in place for early euthanasia base on these observations. One mouse met these criteria 6 weeks after implantation, so all mice were sacrificed at this time point. Two weeks and 6 weeks post iv injection, mice were anesthetized by isoflurane gas inhalation, euthanized by cervical dislocation according to standard guidelines, and then quickly perfused with PBS by intracardiac injection. Mice numbers per group are at W2 n = 6 for NB8pMigr, n = 4 for NB8x4, n = 5 for NB8x7, and NB8x4x7, while at W6 n = 5 for all mice groups. Pieces of liver, lung, adrenal gland, heart and kidneys were either snap-frozen in liquid nitrogen and kept at -80°C until RNA extraction, or formalin-fixed for 12h before processing, and paraffin-embedding.

### Bone Marrow extraction

At sacrifice, femurs and tibias of NSG mice were cut with scissors, and muscles were rubbed off. BM was then flushed out with PBS using a 25G needle. After 10 min centrifugation at 10’000 rpm, BM was fully disaggregated in PBS using a 19G needle. Erythrocytes were lysed using Red Blood Cell Lysis Buffer (Biolegend) for 10 min at 37°C. BM was washed two times in PBS, and dry pellet was kept at -80°C until RNA extraction.

### Total RNA extraction

Total RNA from cell lines and mouse BM were extracted using the RNeasy Mini Kit (Qiagen, Hilden, Germany) according to manufacturer’s instructions. For total RNA extraction from murine organs, 30–50 mg pieces of snap-frozen tissues were disrupted and homogenized in TRIzol Reagent (Invitrogen) for up to 15s using a Polytron PT 1200E (BLANC-LABO, Switzerland). Total RNA was then extracted using the miRNeasy Mini Kit (Qiagen) according to manufacturer’s instructions.

### Semi-quantitative real-time PCR

Total RNA was reverse-transcribed using PrimeScript RT reagent Kit, according to the manufacturer’s instructions (TAKARA Bio Inc., Shiga, Japan). Semi-quantitative real-time PCR was performed using Corbett Rotor-Gene 6000 (Qiagen) with QuantiFast SYBR Green PCR Kit (Qiagen) to analyze *eGFP* expression levels using primer specific for *eGFP*: *fw* 5’- ACT ACC AGC AGA ACA CCC C -3’ and *rev* 5’- TCA CGA ACT CCA GCA GGA C -3’. Cycling condition were 5min at 95°C, 40 cycles of 10s at 95°C, 30s at 60°C, 1s at 72°C. EGFP expression levels in mouse tissues were assessed according to the standard curve method.

### Immunohistochemistry

Hematoxylin and eosin (H&E) and IHC stainings were performed at the Mouse Pathology Facility of Lausanne University (Epalinges, Switzerland). IHC labeling was performed using the mouse monoclonal anti-human vimentin Ab (clone RV202, GeneTex, CA, USA), anti-Ki67 (clone MIB-1, Dako, Denmark), and anti-cleaved caspase-3 (ASP175)(#9661, Cell Signaling Technology Inc.) on 4 μm-thick paraffin-embedded mouse tissue sections.

### Statistical analyses

Statistical analyses were performed using GraphPadPrism 6.0 (GraphPad Software Inc., San Diego, CA, USA). *p<0.05 represented significance; **p≤0.01 and ***p≤0.001 were interpreted to be highly significant.

## Results

### CXCR4 and CXCR7 receptors impact on *in vitro* invasion capacity of NB cells

**Fig 1:**
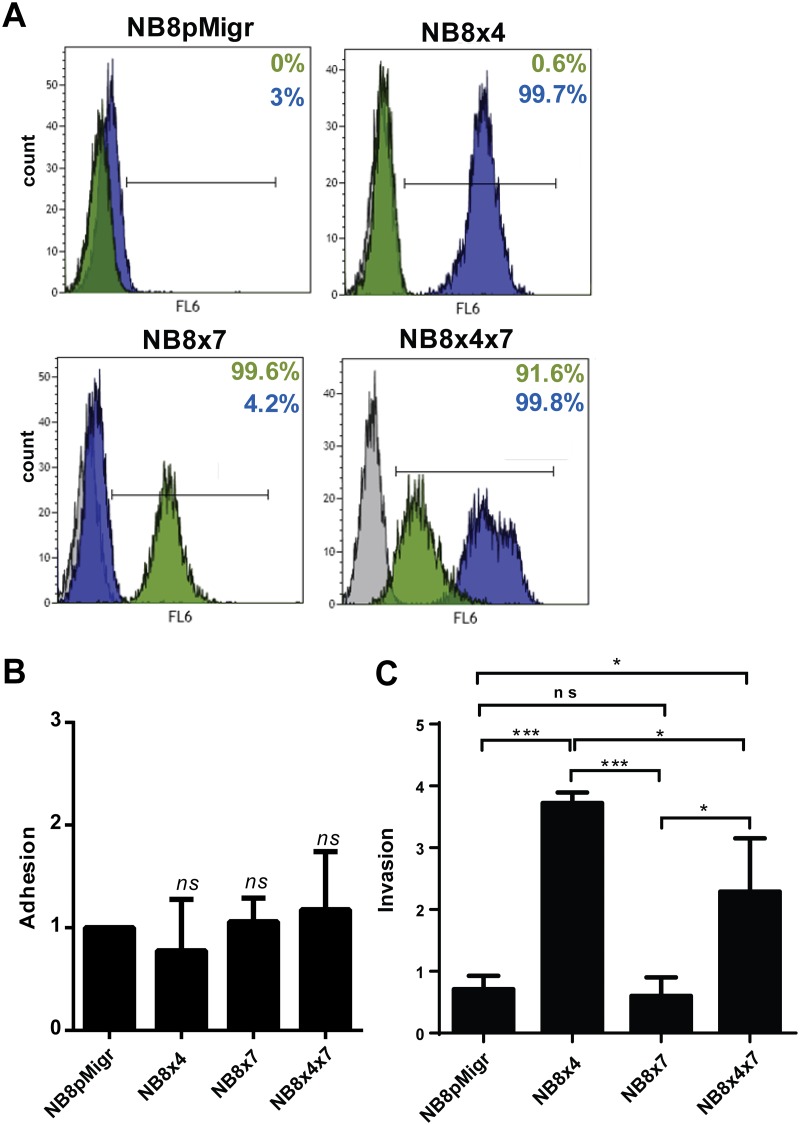
CXCR4 and CXCR7 receptors modulate invasive, but not adhesive properties of transduced NB cells *in vitro*. **A.** CXCR4 and CXCR7 cell surface expression in the NB8 transduced cell lines. CXCR4, CXCR7 or a combination of both CXCL12 receptors was overexpressed in the IGR-NB8 cell line by retroviral infection. NB8pMigr cell line represents control cells transduced with the pMigr empty vector. Percent of CXCR4 (blue) and CXCR7 (green) positive transduced cells are indicated. Grey histogram: cells stained without primary antibodies; blue and green histograms: cells stained with anti-CXCR4 and anti-CXCR7, respectively. **B**. Adhesion capacities of NB8x4, NB8x7, NB8x4x7, and the mock transduced NB8pMigr control cell lines on HUVEC monolayer *in vitro*. Mean ± SD of the ratio of adherent NB cell number relative to that of NB8pMigr control cells are plotted in the graph. Five independent experiments were conducted in triplicates. **C.** Invasive capacities of transduced NB8 cells toward CXCL12 through Matrigel. Mean ratio ± SD of the number of invasive cells in presence versus in absence of CXCL12 are plotted in the bare graph. Three independent experiments were conducted in triplicates. Multiple comparisons were performed using the parametric one way Anova test with Bonferroni corrections: *p<0.05, ***p<0.0005.

To investigate the respective role of CXCR4 and CXCR7 receptors on the homing and invasive phases of the metastatic process, we used transduced variants of the human IGR-NB8 cell line, mock transfected (NB8pMigr) or stably overexpressing individual CXCR4 (NB8x4), CXCR7 (NB8x7), or a combination of both receptors (NB8x4x7), described previously [35] (Fig 1A).

The ability of these transduced NB cells to adhere on endothelial cells (HUVEC) was first analyzed *in vitro*. Neither CXCR4 nor CXCR7 were capable to influence NB cells adhesion capacities on HUVECs when overexpressed in the IGR-NB8 cell line, both in absence (Fig 1B) or in presence of CXCL12 (data not shown).

We next investigated whether *in vitro* invasive properties of NB cells were modified by the expression of any of the two CXCL12 receptors. Overexpression of CXCR4 alone resulted in a significant increase of the *in vitro* invasive potential of NB8x4 cells toward the chemokine CXCL12, relative to NB8pMigr control cells (Fig 1C). In contrast, exogenous expression of CXCR7 had no effect on NB8 invasive property. Interestingly, the invasive capacity of NB8x4x7 cells was enhanced relative to NB8x7 cells, but was reduced when compared to NB8x4 cells (Fig 1C), highlighting a crosstalk between both CXCL12 receptors *in vitro*.

### CXCR4 and CXCR7 receptors are involved in site-specific homing of circulating NB cells

To focus our study in the specific homing phase of metastatic process, we developed an *in vivo* tail vein injection model allowing evaluation of site-specific metastatic seeding of tumor cells, while avoiding tumor cells exit from their primary site of growth. Thus, to investigate whether expression of any one of the two CXCL12 receptors or both in NB tumor cells could influence their selective homing, eGFP^+^ transduced IGR-NB8 control cells or cell lines overexpressing either CXCR4, CXCR7, or combined receptors, were injected iv in NSG mice. Mice were sacrificed 2 weeks (W2) and 6 weeks (W6) after iv injections for investigation of specific organ invasion patterns of tumor cells, and for tumor growth evaluation.

**Fig 2:**
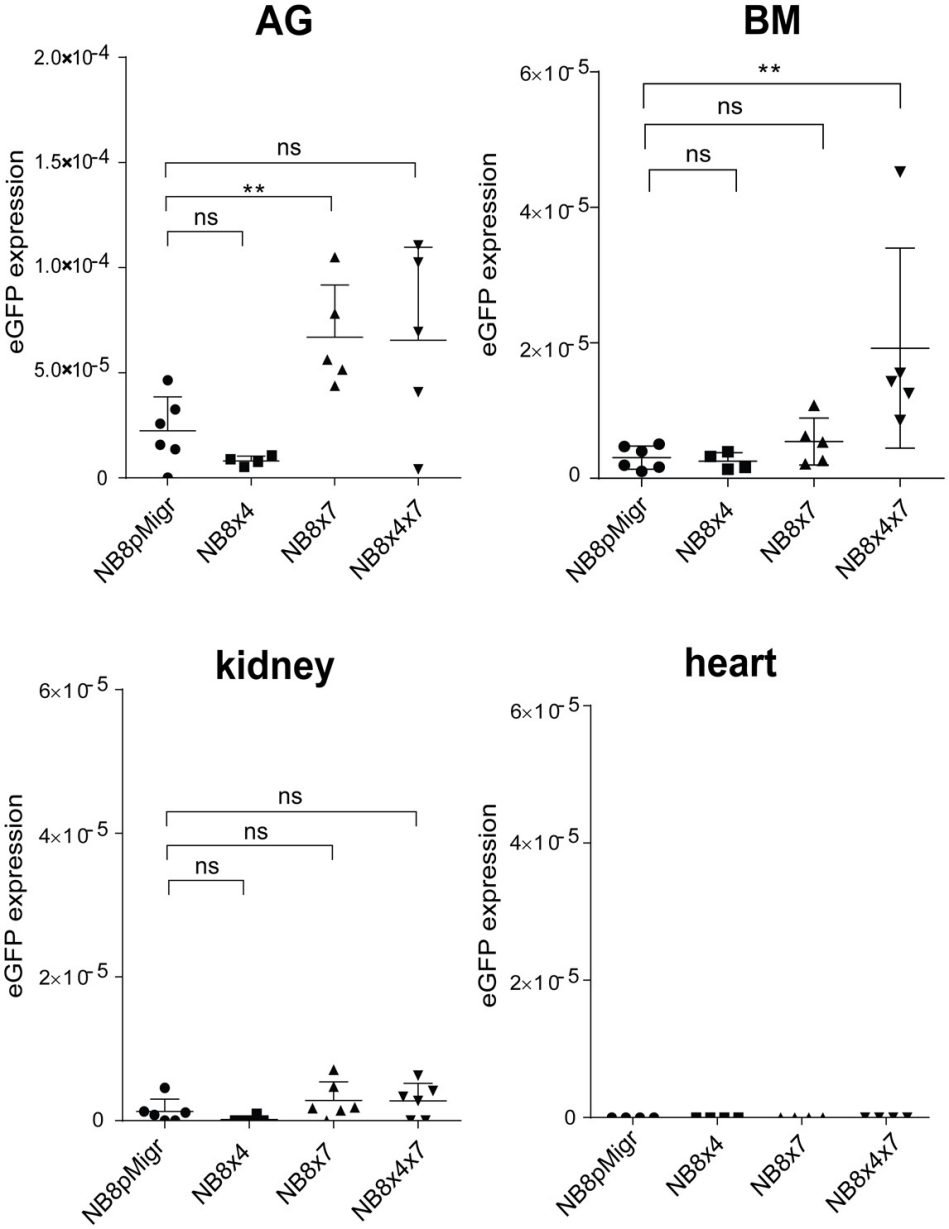
Organ-specific involvement of CXCR7 and CXCR4 in NB cell homing. Analyses of microscopic metastases derived from iv injection of NB8pMigr, NB8x4, NB8x7, and NB8x4x7 cells in NSG mice. *eGFP* mRNA expression levels measured by semi-quantitative real-time PCR analyses in adrenal gland (AG), bone marrow (BM), kidney, and heart of all mice per groups at week 2 post injection. At least two independent qPCR analyses were conducted per organ and per mouse. Individual values, and mean values ± SD of *eGFP* mRNA expression levels detected in transduced NB8 cell lines are plotted in individual graphs for each organ (Mann-Whitney test **p<0.01).

At W2, no macroscopic metastases could be observed at dissection after detailed examination of mice. Microscopic dissemination patterns of transduced eGFP^+^-IGR-NB8 cell lines in liver, AG, BM, lungs, kidney, and heart were first measured by real-time PCR for the detection of eGFP mRNA expression. These analyses revealed significant eGFP signals in the AG, the BM and the liver derived from all transduced IGR-NB8 cells (Fig 2 and data not shown). In contrast, a very weak eGFP signal was measured in the kidney and the lungs of mice injected with all transduced cell lines (Fig 2 and data not shown). No eGFP expression was detected in the heart of mice injected with control or CXCL12-receptor expressing cells (Fig 2), indicating that, at W2, circulating NB cells do not invade such organ, even in presence of chemokine receptors. In addition, these investigations revealed that IGR-NB8 cells overexpressing CXCR7 (NB8x7) displayed an enhanced capacity to invade AG, when compared to NB8pMigr control cells (Fig 2), suggesting that specific homing to the AG could be mediated by CXCR7 expression. Moreover, while neither CXCR4 nor CXCR7 displayed any significant effect on NB cell homing to the BM, a strongly enhanced eGFP signal was observed in the BM of NB8x4x7 group of mice (Fig 2), suggesting a positive effect of the coexpression of CXCR4 and CXCR7 receptors on NB cell dissemination to the BM.

**Fig 3:**
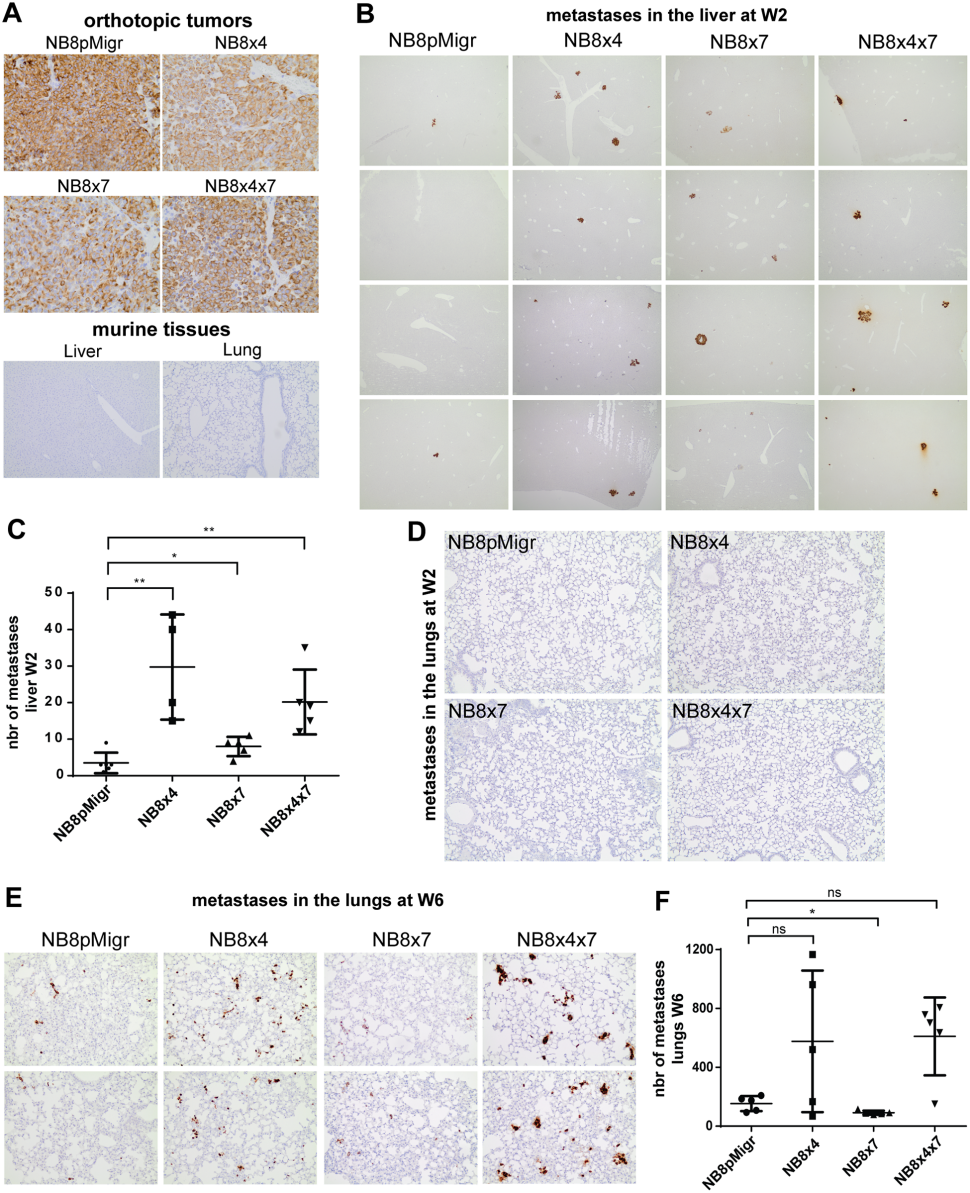
CXCR7 and CXCR4 favor site-specific homing of circulating NB cells after iv injection. **A.** Vimentin IHC staining of paraffin-embedded sections of orthotopic primary tumors derived from NB8pMigr, NB8x4, NB8x7, and NB8x4x7 cells (magnification 40x) [35], and normal lung and liver tissues from NSG mice (magnification 10x). **B.** One representative picture of vimentin IHC staining of liver tissue sections from 4 mice per group at W2 post iv injection (magnification 4x). **C.** Graph representing individual numbers, and mean numbers of hepatic metastases per group at W2 ± SD. Tumor foci were counted under light microscope in two complete liver tissue sections per mouse (Mann Whitney test, *p<0.05, **p<0.005). **D.** Vimentin IHC staining of one representative lung tissue sections per group at W2 post injection (magnification 10x). **E.** Vimentin IHC staining of representative lung tissue sections from two mice per group at W6 post injection (magnification 20x). **F.** Graph representing numbers of vimentin positive micrometastases in lungs for each mouse, and mean numbers per group ± SD at W6 post injection. Vimentin-positive micrometastases were counted in 11 randomly selected fields (magnification 10x) of one section of paraffin-embedded lung tissue of five mice per group (Mann Whitney test, *p<0.05).

In contrast to BM and AG organs where the eGFP expression analysis concerned the totality of the organ, only representative samples of larger organs, such as liver and lungs, could be analyzed by real-time PCR. Therefore, for more complete and precise examination of micrometastases dissemination patterns in those organs, large tissue sections were examined by IHC. Anti-human vimentin labeling was selected as it gave strong and highly specific signals in NB tumors, as shown in orthotopic NB tumors derived from all transduced IGR-NB8 cells [35], while normal murine tissues were negative (Fig 3A). At W2, anti-vimentin IHC staining revealed that the number of hepatic micrometastases detected in NB8x4, NB8x7, and NB8x4x7 groups was increased as compared to the control NB8pMigr group (Fig 3B and 3C), suggesting that both CXCL12 receptors are involved in NB cell homing to the liver.

Although the lungs do not represent a common metastatic site in NB, they may also be involved and were thus investigated. At W2, almost no metastatic NB cells could be detected by vimentin IHC (Fig 3D), which is in accordance with the very low eGFP signal detected by real-time PCR (data not shown). Thus, micrometastases dissemination in the lungs was further explored at W6 by IHC, which revealed the presence of numerous microscopic metastases or single NB cells at this time point (Fig 3E and 3F). These data indicate a delay in the occurrence and growth of micrometastases in the lungs compared to the AG, the BM and the liver. Although not statistically significant due to the weak mice number (p = 0.413 for NB8x4 and p = 0.056 for NB8x4x7), the amount of vimentin-positive micrometastases in lungs is increased in the NB8x4 and NB8x4x7 groups as compared to NB8pMigr control group (Fig 3F), suggesting a potential role for the CXCR4 receptor in NB cell dissemination into the lungs. Surprisingly a weak decrease in metastatic numbers was observed in the NB8x7 group compared to NB8pMigr group, suggesting that CXCR7 might not favor NB cell homing to the lungs (Fig 3F).

**Fig 4:**
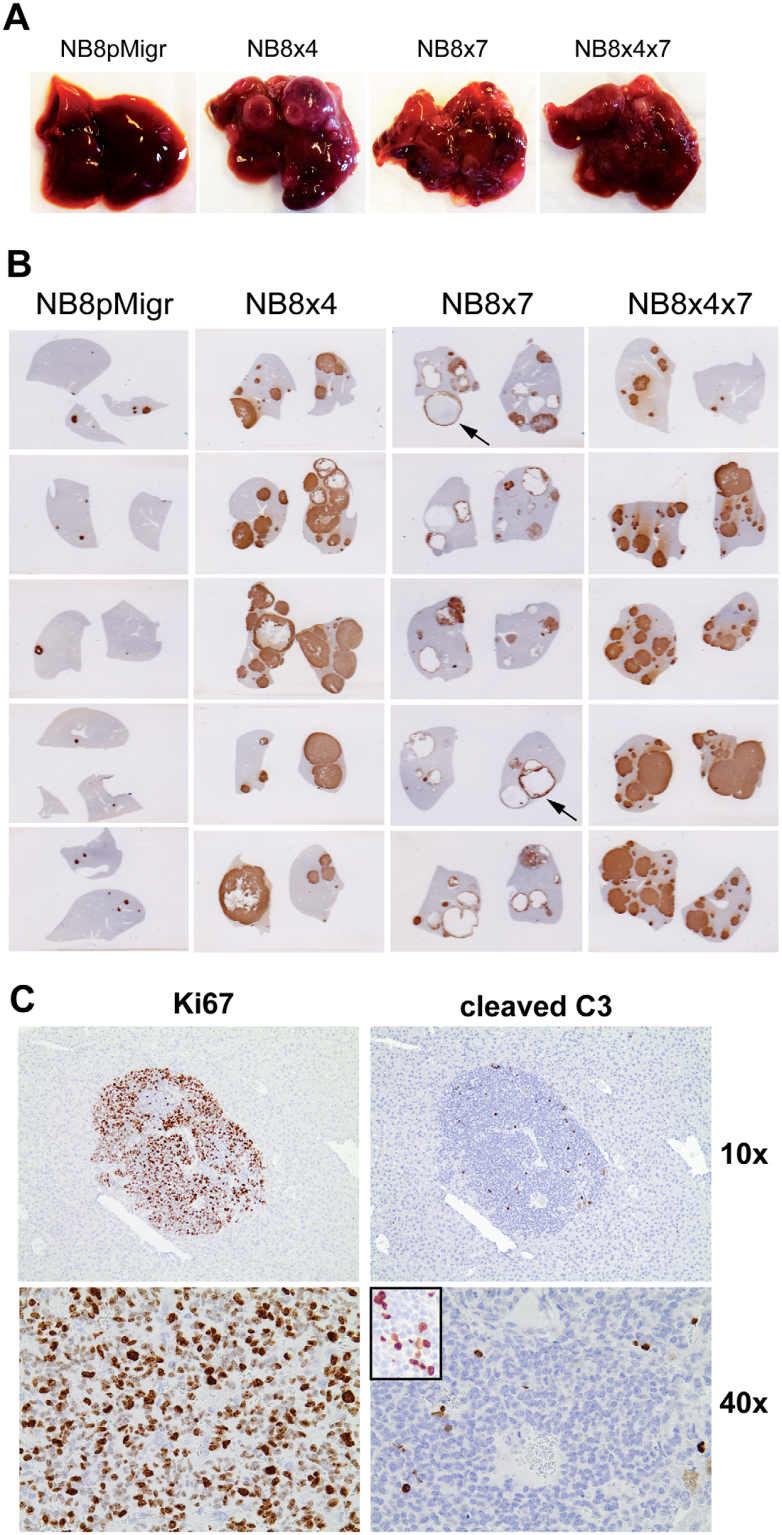
CXCR4 and CXCR7 receptors enhance metastatic growth in the liver. **A.** Pictures of one representative liver of each group at sacrifice (W6). **B.** Vimentin IHC staining of liver sections of 5 mice per group at W6. **C.** Ki67 and cleaved caspase-3 (C3) IHC of one representative hepatic metastasis at W6 (magnifications 10x and 40x). Positive control for cleaved C3 is inserted in the small insert (NB8x4 orthotopic tumor, magnification 40x).

Examination of mice at W6 confirmed the massive invasion of the liver (Fig 4), the bone marrow and the adrenal glands (data not shown), while the lungs are only involved with microscopic metastases (Fig 3E).

Altogether these results demonstrate that CXCR4 and CXCR7 receptors are both involved in NB cell homing, however their involvement may vary in an organ-dependent manner.

### CXCR4 and CXCR7 receptors enhance metastatic growth in the liver

We next investigated whether and how both CXCL12 receptors influence tumor growth, once disseminated to target organs. As macroscopic metastases were only observed in the liver at W6 (Fig 4A), our investigations were focused on this organ. Indeed, livers from mice implanted with NB8x4, NB8x7, and NB8x4x7 cells displayed numerous macroscopic metastases at W6, in contrast to the NB8pMigr control group, which did not present any liver macroscopic metastases (Fig 4A). The detailed analysis of hepatic macrometastases by IHC at W6 confirmed their strongly increased size in NB8x4, NB8x7, and NB8x4x7 groups, as compared to the NB8pMigr control group (Fig 4B). Hepatic metastases displayed almost no stroma infiltration. Interestingly, liver metastases derived from NB8x7 cells displayed massive hemorrhage, resulting in the formation of large blood lake (Fig 4B, black arrows).

Strong Ki67 staining and sporadic cleaved-caspase-3 staining by IHC further revealed an extremely high proliferation index together with the presence of only rare apoptotic cells in hepatic metastases of all mice groups (Fig 4C), indicating that hepatic metastases at W6 are mainly composed of highly proliferative cells. Interestingly, high expression level of CXCL12 was previously observed in the liver in mice [12,13]. Overall, these data suggest an implication of the CXCR4/CXCR7/CXCL12 axis in hepatic metastases growth induction in such iv model.

## Discussion

Our previous *in vivo* orthotopic studies in NB revealed an essential growth-promoting role for CXCR4, the long-time known CXCL12 receptor, which could be modulated by co-expression of CXCR7, the second and more recently identified CXCL12 receptor [35]. Such observations already indicated a possible cooperation or competition between the two receptors in NB. However in this orthotopic model, in contrast to several reports in different cancers, including NB [16], CXCL12 receptors either alone or in combination did not increase metastatic dissemination nor modified the typical NB metastatic pattern, suggesting that these chemokine receptors may not influence NB metastatic process [13,35]. Nevertheless, in such NB orthotopic model, the very high level of CXCL12 chemokine present in the adrenals [12,13], may have mobilized CXCR4/7 receptors-expressing cells, and thus prevented their dissemination.

In this context, we were interested in specifically dissecting CXCR4/CXCR7/CXCL12 axis involvement in the specific homing phase of metastatic dissemination. As preliminary *in vitro* investigations, we observed that adhesive properties of NB cells to HUVEC were not influenced by expression of any CXCL12 receptors, in presence or in absence of the ligand. These observations differ from previous studies in other tumor models showing that CXCR7 expression improved tumor cell adhesion on HUVECs in presence, and in absence of chemokine ligands [22,29,38,39]. In addition, we observed that CXCR4 expression alone significantly enhanced NB cells *in vitro* invasive capacity, while CXCR7 alone had no effect, confirming a previous report in NB [34]. However, the observed modulation of *in vitro* CXCR4-mediated invasive properties by CXCR7, that was not detected in this earlier report [34], further supports an existing interaction and/or competition between both receptors as previously described in various cancers [13,30,31,33,35].

To investigate whether and how the CXCR4 and CXCR7 receptors are involved in metastatic cell homing and invasion into specific NB target organs, we worked out an *in vivo* iv model, where control and transduced NB cells, expressing either one or both receptors, were implanted directly into the circulation of immunodeprived NSG mice. In such conditions, macroscopic tumors could only be observed in the liver at W6. Nevertheless, a more sensitive detection of NB cells in mice organs either by real-time PCR, or by vimentin staining, revealed the presence of NB cells in different organs. Although in absence of CXCL12 receptors expression, circulating NB8pMigr control cells preferentially home to the AG, the liver and the BM, already 2 weeks after NB cells implantation; CXCR4 and/or CXCR7 expression potentiate NB cell homing in an organ-dependent manner. In all mice groups, no eGFP signal was detected in the heart, while extremely low eGFP signals were detected in the lungs and the kidneys at W2. Metastatic dissemination of NB cells in the kidney had been previously described in iv and subcutaneous models of NB, although this organ is not targeted by NB cells in patients [16]. Our observations thus indicate that NB cells are able to preferentially home and establish in specific organs.

Interestingly, CXCR4 expression enhanced NB8 cell implantation in the liver and potentially in the lungs, while CXCR7 expression promoted NB cell homing to the AG and the liver. Moreover, coexpression of CXCR4 and CXCR7 significantly increased NB cell homing to the BM, while neither CXCR4 nor CXCR7 alone played a significant role in the BM. Such effect resulting from the coexpression of CXCR4 and CXCR7 could not be observed in the other organs analyzed. Such enhanced tumor cell specific homing in the BM mediated by coexpression of both CXCR4 and CXCR7 receptors correlate with a previous study reported in a rhabdomyosarcoma model [38].

Thus, CXCR4 and CXCR7 receptors influence NB cell homing and dissemination in a complex and organ-specific manner. The differences in the involvement of CXCR4 and CXCR7 receptors between *in vitro* and *in vivo* NB cell invasion can be explained by the fact that *in vivo* invasion results from an environment-dependent multistep process, involving the ability of tumor cells to arrest in a specific organ, adhere to endothelial cells, extravasate, and establish an appropriate microenvironment for proliferation. This is the first study reporting an organ-specific influence of balanced expression of CXCR4 and CXCR7 chemokine receptors in metastatic dissemination. Indeed, studies in other tumor models revealed either a role for CXCR4, but not for CXCR7, in metastases induction [40], or the enhanced metastatic potential conferred by CXCR7 [39].

In this iv model, AG, which corresponds to the natural and privileged site for primary NB, reveals as an early site of invasion, even by CXCR4/7-negative NB8pMigr cells. However, CXCR7 in contrast to CXCR4 was significantly involved in enhancing AG selective homing. Interestingly, controlateral AG invasion has not been reported in patients with metastatic NB. Therefore, CXCR7 is a possible player not only in metastatic homing of NB cells, but also in other mechanisms controlling NB development in the AG, which deserves to be further investigated.

In addition, we observed that the lungs were generally involved with only very small micrometastases at W6, likely to reflect a less attracting and/or less permissive environment for NB cells implantation and growth. This is in accordance with the infrequent clinical occurrence of NB metastatic spread in the lung, which represents late event in widely disseminated metastatic disease [41,42]. The role of CXCR7 in mediating lungs metastases vary according to cancer types [39,43–45]. In this iv NB model, CXCR7 expression faintly reduced the number of lung metastases. In contrast, although not statistically significant due to heterogeneity and the low number of mice in this study, CXCR4 receptor (NB8x4 and NB8x4x7) was able to strongly enhance the number of lung metastases, supporting a CXCR4 receptor influence on NB cell invasion of the lungs, and a possible involvement of the CXCR4/CXCL12 axis in lungs metastases occurrence in patient with highly disseminated NB disease.

Examination of murine livers revealed the impact of CXCR4 and CXCR7 receptors both on the selective homing and on macrometastatic growth induction, as both the number and the size of liver metastases were strongly increased with cells expressing CXCR4 and/or CXCR7. Hepatic metastases displayed elevated proliferation index together with rare apoptotic cells in all groups, confirming that liver is a highly permissive environment for NB cells growth. However, cells expressing CXCR7 alone (NB8x7 cells) generated highly hemorrhagic liver metastases with large blood lakes. Such CXCR7-mediated metastatic pattern may result from distinct and additional mechanisms that need to be further investigated. These data confirm the role of CXCR4 in promoting growth of primary and secondary tumors as already demonstrated [13]. However, this iv model did not confirm the abrogative effect of CXCR7 on CXCR4-mediated tumor growth observed in our orthotopic NB model [35]. These somehow contrasting results may be due to the different models and environments investigated in the orthotopic and iv models.

Altogether these data demonstrate that in this NB model, CXCR4 and CXCR7 receptors play a role in organ selective homing and invasion, which depend on the target organ. The balance in the expression levels of the two CXCL12 receptors reveals their participation into complex mechanisms controlling metastatic homing and growth, encouraging further investigations into the role of these receptors and tumor environment. These findings also reveal CXCR7, in addition to CXCR4, as possible targets for new therapeutic strategies against aggressive and metastatic NB disease.
